# Unusual blue discolouration of the ascending aorta in a patient with Churg–Strauss syndrome undergoing surgical aortic valve replacement: a case report

**DOI:** 10.1093/jscr/rjag818

**Published:** 2026-09-16

**Authors:** Aditya Gaur, David Rose, Marcus Taylor, Khabab Abbasher Hussien Mohamed Ahmed

**Affiliations:** Yeovil District Hospital, Somerset NHS Foundation Trust, Higher Kingston, Yeovil, United Kingdom; Department of Cardiothoracic Surgery, Blackpool Teaching Hospitals NHS Foundation Trust, Whinney Heys Road, Blackpool, United Kingdom; Department of Cardiothoracic Surgery, Blackpool Teaching Hospitals NHS Foundation Trust, Whinney Heys Road, Blackpool, United Kingdom; Faculty of Medicine, University of Khartoum, Khartoum 11111, Sudan; Department of Pathophysiology, School of Medicine, St. George’s University, St. George's, Grenada

**Keywords:** Churg–Strauss syndrome, aortic valve replacement, blue aorta, systemic vasculitis, cardiac tamponade, case report

## Abstract

Churg–Strauss syndrome (eosinophilic granulomatosis with polyangiitis) is a rare systemic vasculitis that predominantly affects small- to medium-sized vessels, with large-vessel involvement being exceptional. We report a 72-year-old woman with Churg–Strauss syndrome and severe low-flow, low-gradient aortic stenosis (left ventricular ejection fraction 15%) who underwent surgical aortic valve replacement following multidisciplinary Heart Team assessment. Intraoperatively, an unusual blue discolouration of the ascending aortic intima was observed. Postoperatively, the patient developed cardiac tamponade requiring urgent reintervention and had a prolonged recovery before being discharged to rehabilitation. This case expands the spectrum of vascular manifestations in Churg–Strauss syndrome and highlights potential perioperative challenges during cardiac surgery.

## Introduction

Churg–Strauss syndrome (CSS), also known as eosinophilic granulomatosis with polyangiitis (EGPA), is a rare systemic vasculitis characterized by eosinophilic inflammation and necrotizing vasculitis, often associated with asthma [1]. With a prevalence of 10.7 to 14 per million, CSS does not exhibit a gender or ethnic predisposition [2]. While the exact aetiology remains unclear, CSS is widely considered an autoimmune disorder in which eosinophils play a central role in pathogenesis. The disease typically progresses in stages, beginning with asthma or allergic rhinitis, followed by eosinophilia and tissue damage, culminating in a potentially fatal phase of vasculitis. However, the condition can generally be managed effectively with immunosuppressive therapy [3, 4].

The diagnostic criteria for CSS, established by the Chapel Hill Consensus Conference in 1994, emphasize both clinical and pathological findings, including eosinophil-rich granulomatous inflammation of the respiratory tract and necrotizing vasculitis affecting small- to medium-sized vessels, often accompanied by asthma and eosinophilia [5]. CSS can affect multiple organ systems, leading to complications such as peripheral neuropathy, skin lesions, segmental necrotizing glomerulonephritis, and pulmonary infiltrates. Cardiac involvement is relatively frequent — reported in up to ~60% of patients when sensitive imaging is used — and represents a leading cause of disease-related mortality; it may present as conduction disturbances, pericarditis, myocarditis, or valvular disease, the last of which is associated with a poorer prognosis [6–9].

Vascular abnormalities involving larger vessels such as the aorta are rare and poorly documented. This case report presents a unique instance of a patient with CSS undergoing surgical aortic valve replacement (SAVR), during which an unusual blue discolouration of the ascending aorta was noted. To the best of our knowledge, this finding has not previously been reported in the context of CSS. This report aims to discuss the clinical presentation, surgical management, and unusual vascular findings in a patient with CSS undergoing SAVR, along with the potential mechanisms contributing to the blue discolouration and its implications for surgical and medical management in similar cases.

This work was reported in line with the Surgical CAse REport (SCARE) criteria [10].

## Case presentation

A 72-year-old White female of English ethnicity was referred to the cardiothoracic team for evaluation of progressively worsening shortness of breath and fatigue over the preceding year. Initial evaluations excluded asthma and atrial fibrillation. Six months after symptom onset, the patient developed bilateral pedal oedema, prompting a referral to cardiology. Echocardiography revealed significant aortic stenosis (AS), necessitating further surgical assessment.

Her medical history included atrial fibrillation, mild coronary artery disease, hypertension, hypercholesterolaemia, heart failure (New York Heart Association Class II), osteoarthritis, depression, bronchiectasis, type 1 diabetes mellitus, and a myocardial infarction in 2017. In addition, the patient had peripheral neurovasculitis secondary to CSS, managed with prednisolone and mycophenolate mofetil. Her social history revealed that she was an ex-smoker with occasional alcohol consumption.

Cardiovascular examination revealed an audible ejection systolic murmur and bilateral peripheral oedema. Routine blood investigations showed a normal white cell count (5.3 × 10^9^/L) and eosinophil count (0.11 × 10^9^/L), along with the presence of perinuclear antineutrophil cytoplasmic antibodies. Pre-operative echocardiography confirmed low-flow AS with a markedly reduced left ventricular ejection fraction (LVEF) of 15% (Fig. 2), reflecting a significant decline from 50% recorded 10 months earlier. Additional findings included inferior septal hypokinesia, severe AS with an aortic valve area of 0.7 cm^2^, bilateral atrial dilatation, and normal right ventricular function. Coronary angiography revealed mild plaque disease consistent with her history of coronary artery disease following myocardial infarction.

**Figure 1:**
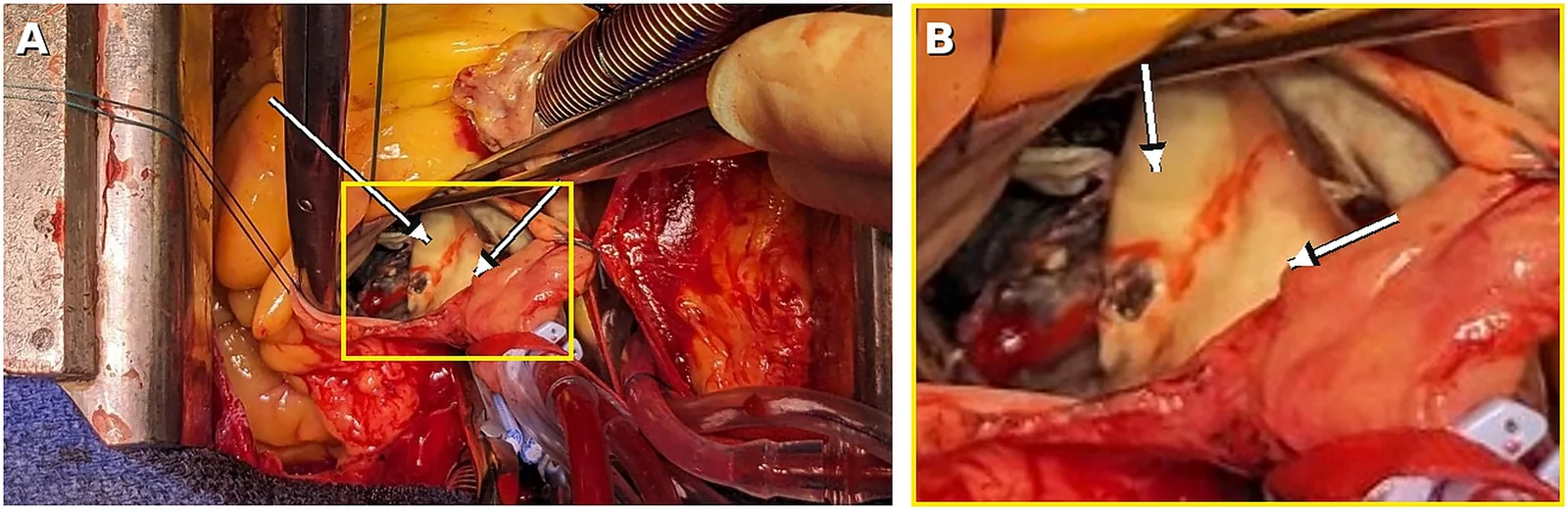
Intraoperative photographs taken during surgical aortic valve replacement in a patient with Churg–Strauss syndrome. (A) Overview of the operative field; the boxed area indicates the region of interest and the arrows point to the intimal surface of the opened ascending aorta. (B) Magnified view of the boxed area, demonstrating the blue/dusky discolouration of the ascending aortic intima (arrows) extending towards the aortic root; the outer adventitia appeared normal. The discolouration was more conspicuous to the operating team in vivo than is conveyed by photography under theatre lighting.

Following multidisciplinary Heart Team review, and after weighing the therapeutic options in light of her age, ventricular function, and systemic vasculitis, the patient was scheduled for SAVR rather than transcatheter aortic valve replacement (the rationale is discussed below). The procedure was performed via a midline sternotomy under general anaesthesia. Cardiopulmonary bypass (CPB) was initiated prior to aortic cross-clamping. During the procedure, an unusual finding was noted: the tunica intima of the ascending aorta displayed a distinct blue discolouration, extending proximally towards the aortic valve (Fig. 1). The tunica externa (adventitia) appeared normal, with no other structural abnormalities identified apart from the discolouration. The discolouration was appreciated directly by the operating team; as is common with intraoperative photography under theatre lighting in a blood-stained field, it is reproduced only subtly in the clinical photograph, and a magnified view is therefore provided to aid appreciation (Fig. 1B). Despite this observation, the procedure proceeded as planned, and a 23 mm Edwards INTUITY Elite rapid-deployment valve (Edwards Lifesciences, Irvine, CA, USA) was successfully implanted without intraoperative complications; this rapid-deployment prosthesis was selected in part to minimise cross-clamp and CPB times given her severely impaired ventricular reserve. Postoperative transoesophageal echocardiography confirmed significant improvement in valve function and LVEF.

**Figure 2:**
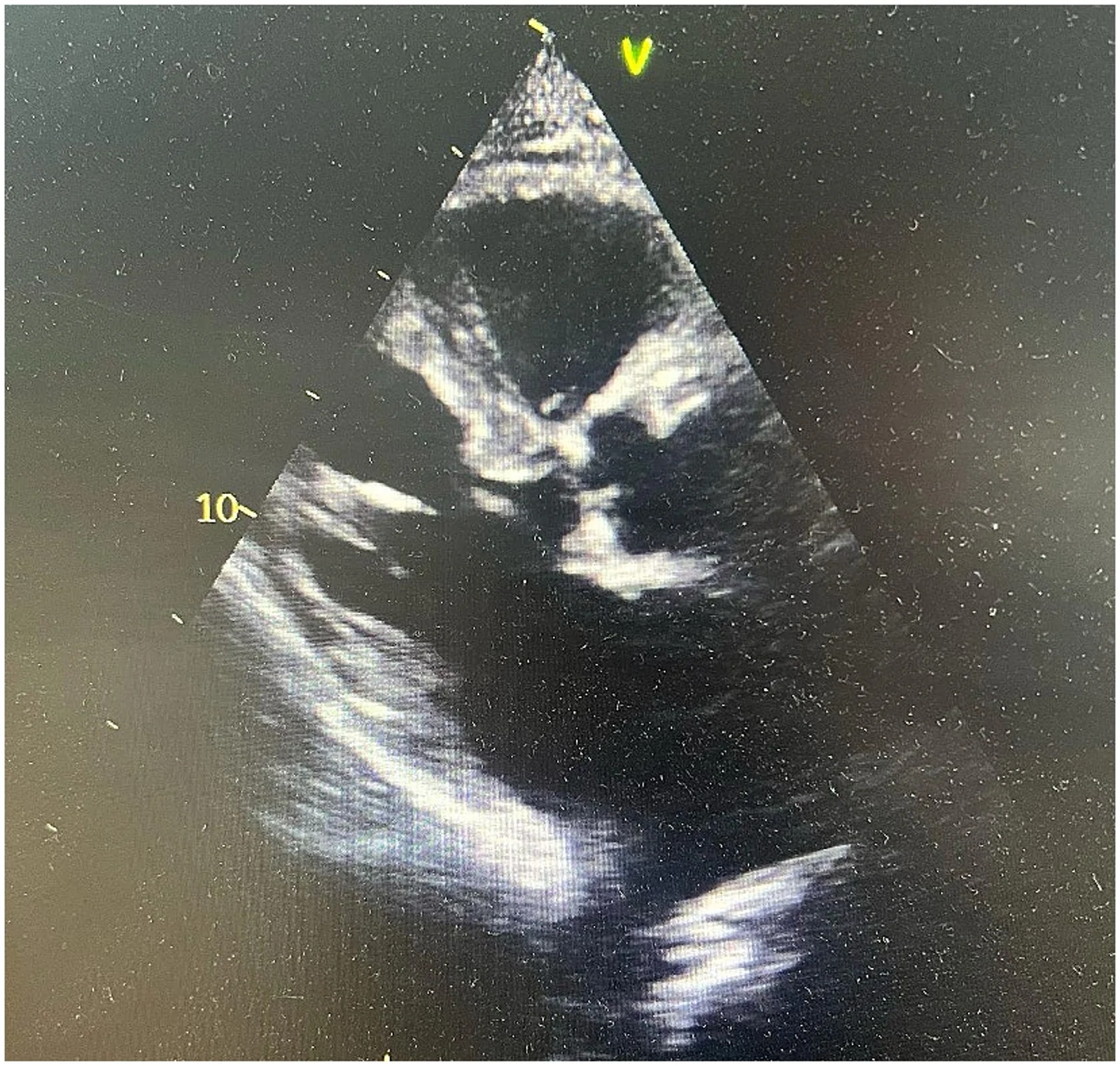
Pre-operative apical transthoracic echocardiographic view demonstrating severe aortic stenosis with markedly impaired left ventricular systolic function (left ventricular ejection fraction 15%).

Postoperatively, the patient was managed in the cardiothoracic intensive care unit (ITU). On day six, she experienced a peri-arrest event requiring emergency surgical intervention; repeat sternotomy revealed cardiac tamponade, which was evacuated. She subsequently remained in the ITU for a total of 34 days before transfer to a local hospital for ongoing rehabilitation. Several factors contributed to this prolonged inpatient course: her severely impaired baseline left ventricular function (LVEF 15%) predisposed her to a low cardiac output state, necessitating prolonged inotropic and vasopressor support with cautious, staged weaning; the day-six cardiac tamponade and re-exploration represented a significant haemodynamic setback; and her underlying CSS, together with chronic immunosuppression (prednisolone and mycophenolate mofetil), increased her susceptibility to postoperative complications and slowed convalescence. The resulting deconditioning required an extended period of supervised inpatient cardiac rehabilitation. No further major complications were observed, and she was ultimately discharged in a stable condition.

To our knowledge, blue discolouration of the ascending aortic tunica intima has not previously been documented in a patient with CSS undergoing SAVR; the following discussion considers its possible mechanisms, its potential impact on surgical outcomes, and the wider clinical implications.

## Discussion

This case report highlights a rare and previously undocumented finding of blue discolouration of the ascending aorta in a patient with CSS undergoing SAVR. While CSS predominantly affects small- to medium-sized vessels, large-vessel involvement, such as the aorta, is exceedingly rare and sparsely reported in the literature. Such abnormalities are more commonly associated with other forms of vasculitis, such as Takayasu arteritis or giant cell arteritis [11]. Known cardiac manifestations of CSS include myocarditis, pericarditis, and conduction disturbances [6–9].

The aetiology of the blue discolouration in this case is unclear but may be attributed to chronic vascular inflammation associated with CSS [12]. Alternative explanations include vascular injury secondary to immune complex deposition, a mechanism observed in other vasculitides such as hepatitis B-associated vasculitis but less commonly in CSS [13]. Haemosiderin deposition, resulting from chronic inflammation and vascular damage, is another plausible hypothesis, as it has been reported in patients with CSS [14]. Although this discolouration did not alter the surgical approach, it raises important concerns about the structural integrity of the affected vessel and the potential risks for future complications.

Valvular involvement in CSS is uncommon but can occur, with mitral regurgitation being the most frequently reported. Although aortic stenosis is less typical, this case demonstrated its accelerated progression, with the patient’s ejection fraction dropping significantly (from 50% to 15%) over just 10 months. This underscores the potential for CSS to rapidly compromise cardiac function, emphasizing the importance of timely intervention [15].

The choice between SAVR and transcatheter aortic valve replacement (TAVR) in this patient warrants comment. Although TAVR is now an established option across the spectrum of surgical risk, contemporary guidelines recommend an individualized, multidisciplinary Heart Team decision incorporating age, anticipated longevity, prosthesis durability, anatomy, and comorbidity [16, 17]. While a transcatheter approach avoids cardiopulmonary bypass — a theoretical advantage in a patient with severely reduced ejection fraction — several considerations favoured surgery in this case. Her age (72 years) lies at the younger end of the population in whom the durability and long-term outcomes of a surgical bioprosthesis remain advantageous. She presented with low-flow, low-gradient severe AS and severely depressed systolic function, a subgroup in which confirmation of true-severe stenosis and haemodynamic optimization are essential and in which management is complex irrespective of modality. Most importantly, her systemic vasculitis with documented peripheral neurovasculitis raised concern about the suitability and integrity of the peripheral vasculature for large-bore transfemoral access, and about the aortic wall itself — a concern subsequently reinforced by the unexpected intraoperative findings. Accordingly, after Heart Team discussion, surgical replacement was undertaken, and a rapid-deployment prosthesis was selected to limit cross-clamp and bypass times and thereby mitigate the risks posed by her poor ventricular reserve. This case illustrates that, even in the transcatheter era, surgical aortic valve replacement remains an appropriate and sometimes preferable strategy in selected patients with complex systemic disease.

Postoperative cardiac tamponade was a notable complication in this case. While no intraoperative abnormalities were observed, patients with CSS are predisposed to pericardial effusion and tamponade owing to chronic pericardial inflammation and vascular fragility, particularly in the postoperative period [18]. The scarcity of data on perioperative complications in CSS makes it difficult to predict or manage these events. A similar case in the literature describes a patient with CSS undergoing aortic valve replacement who developed a third-degree atrioventricular block, further highlighting the need for vigilance in such cases [19].

This case has several limitations. As a single-case report, its generalizability is limited, and definitive evidence is lacking to fully elucidate the mechanisms underlying the observed aortic discolouration or the ways in which CSS may complicate cardiac surgery. Notably, no aortic tissue was available for histopathological examination: the operative technique (transverse aortotomy with valve replacement) does not entail resection of the aortic wall, so no specimen could be submitted, and the aetiology of the discolouration therefore could not be confirmed histologically. In addition, only a single intraoperative photograph was obtained, and the discolouration — although clearly appreciated *in vivo* — is reproduced only subtly under operative lighting; an annotated, magnified panel has been included to assist interpretation (Fig. 1). Further research and documentation of similar cases, ideally with histological correlation, are crucial to better understand these phenomena and to inform the surgical and medical management of patients with CSS.

## Conclusion

This case highlights the novel finding of blue discolouration of the ascending aorta in a patient with CSS, expanding the understanding of its vascular manifestations. While the exact mechanism remains speculative, the findings suggest potential roles for chronic inflammation, immune complex deposition, or haemosiderin accumulation. The occurrence of postoperative cardiac tamponade further emphasizes the vascular fragility associated with CSS and the need for careful perioperative management. This case underscores the importance of further research to elucidate the pathophysiology of such findings and to improve outcomes in patients with CSS undergoing cardiac surgery.

## Data Availability

Data availability is not applicable to this article, as no new data were created or analysed in this study.
